# Multimodal Limbless Crawling Soft Robot with a Kirigami Skin

**DOI:** 10.34133/cbsystems.0301

**Published:** 2025-06-09

**Authors:** Jonathan Tirado, Aida Parvaresh, Burcu Seyidoğlu, Darryl A. Bedford, Jonas Jørgensen, Ahmad Rafsanjani

**Affiliations:** ^1^ SDU Soft Robotics, Biorobotics Section, The Maersk McKinney Moller Institute, University of Southern Denmark, Odense 5230, Denmark.; ^2^ Drawstring Origami Ltd., London, UK.

## Abstract

Limbless creatures can crawl on flat surfaces by deforming their bodies and interacting with asperities on the ground, offering a biological blueprint for designing efficient limbless robots. Inspired by this natural locomotion, we present a soft robot capable of navigating complex terrains using a combination of rectilinear motion and asymmetric steering gaits. The robot is made of a pair of antagonistic inflatable soft actuators covered with a flexible kirigami skin with asymmetric frictional properties. The robot’s rectilinear locomotion is achieved through cyclic inflation of internal chambers with precise phase shifts, enabling forward progression. Steering is accomplished using an asymmetric gait, allowing for both in-place rotation and wide turns. To validate its mobility in obstacle-rich environments, we tested the robot in an arena with coarse substrates and multiple obstacles. Real-time feedback from onboard proximity sensors, integrated with a human–machine interface, allowed adaptive control to avoid collisions. This study highlights the potential of bioinspired soft robots for applications in confined or unstructured environments, such as search-and-rescue operations, environmental monitoring, and industrial inspections.

## Introduction

Earthworms can crawl on the ground and burrow underground without the use of limbs. For limbless locomotion on flat surfaces, the absence of push points over the surface requires the coordination of body deformation and static friction to generate propulsive forces. The rhythmic contraction of earthworms’ muscles produces peristaltic waves along their slender bodies [1] while friction-enhancing bristles on their skin, called setae, ensure a firm grip on the ground with each stride [2,3]. The setae generate a directionally asymmetric friction that is easy to overcome in the direction of movement but strong enough to prevent sliding back. Thus, 3 fundamental elements of limbless locomotion on terrains with uniform roughness are large deformability, rhythmic contractions, and asymmetric friction. The limbless locomotion of earthworms has inspired the development of several crawling soft robots that replicate some of their morphological features, enabling them to crawl on uniform terrains [4–6], inside pipes [7–9], and through granular media [10,11]. However, unifying all of these in a crawling robot remains unexplored. Additionally, many earthworm-inspired soft robots can only move in a straight line and do not possess steering capabilities, which limits their applicability to unstructured real-world terrains.

To replicate body deformation, several researchers have developed worm-inspired soft robots powered by various actuation mechanisms. For example, Seok et al. [12] developed a peristaltic soft robot powered by shape memory alloy nickel titanium coils integrated into an elastic tubular braided mesh to generate antagonistic axial and radial contractions for rectilinear locomotion on flat surfaces. Das et al. [13] developed a modular soft robot for locomotion in multi-terrain environments using a fluid-driven peristaltic soft actuator capable of 2 active configurations by alternating between positive and negative pressure inputs, producing longitudinal forces for axial penetration and radial forces for anchorage through bidirectional deformation of its central bellows-like structure. Yoon et al. [14] created an untethered soft earthworm robot that operates based on the thermally controlled gas–liquid phase transition of a soft thermoelectric pneumatic device. Calderón et al. [15] developed a pneumatic soft robotic system consisting of 2 expanding ends connected to an extending actuator, replicating the motion and functionality of a single burrowing earthworm segment, enabling locomotion inside horizontal, inclined, and vertical pipes. Zhang et al. [16] further advanced this design by incorporating 3 chambers in the central actuator for navigation and imaging through multi-branch tubular phantoms.

To incorporate asymmetric friction, researchers integrated passive hooks and bristles [13,17,18], multi-material casted setae [19], and 3-dimensional (3D)-printed scales [20] into soft actuators, creating low friction in the movement direction and high friction in the backward direction, thereby enabling 2-anchor crawling [21]. Kirigami is a traditional papercraft technique that enables the transformation of thin, flat sheets into complex 3D structures through patterned cuts, offering functional and flexible skins for crawling robots. An effective strategy for introducing friction asymmetry in soft crawling robots involves covering soft actuators with kirigami skins created by introducing asymmetric repetitive cut patterns into plastic films, where the elongation of the underlying actuator triggers directionally aligned pop-ups [22]. These kirigami skins have enabled bioinspired soft robots to achieve rectilinear locomotion [23,24], burrow through cohesive soil [11] and move by lateral undulation [25]. However, while such stretchable kirigami skins coordinate body deformation with friction asymmetry, existing designs are often limited to uniaxial movement because bending-induced compressive forces during robot steering result in nonuniform and unpredictable deformation of the kirigami skins.

**Fig. 1.**
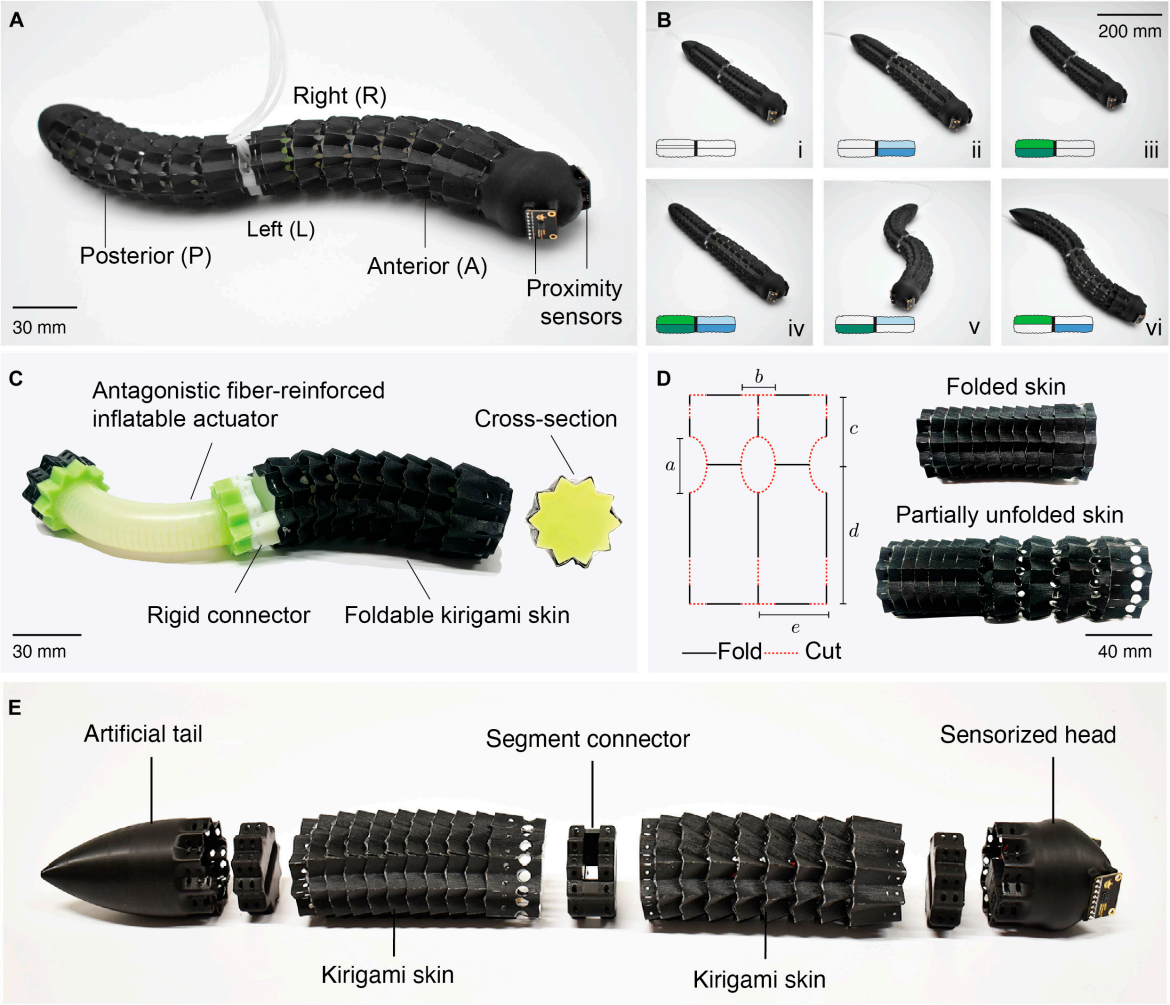
Multimodal crawling robot with kirigami skin. (A) Robot’s body featuring anterior and posterior segments and 2 proximity sensors mounted on its head. (B) Demonstration of different deformation modalities and schematic of inflated chambers. (C) The inner structure of the robot’s body showing the assembly of the foldable kirigami skin and the antagonistic fiber-reinforced inflatable actuator. (D) Geometry of the skin unit cell with black fold lines and red cut lines, and snapshots of the multistable foldable kirigami skin in folded and partially unfolded configurations. (E) External components of the robot, including 3D-printed connectors between the anterior and posterior segments, the kirigami skin, the tail, and the sensorized head housing 2 lateral proximity sensors.

In this work, we propose a limbless crawling soft robot comprising a soft, deformable body made of pneumatic artificial muscles and covered with a foldable kirigami skin designed to break friction symmetry while maintaining high conformability to various combinations of longitudinal and transverse deformations (see Movie S1). The robot is driven by an external pressure source with oscillatory pressurization generated by a central pattern generator (CPG), which can produce stable rhythmic patterns with only simple input signals [26]. Equipped with a rounded head bearing 2 proximity sensors and a pointy tail, the robot can crawl on surfaces with moderate roughness levels and avoid obstacles through assisted teleoperation (see Fig. 1A). This design demonstrates how the synergy between longitudinal and transverse body deformation and friction modulation enables crawling and maneuvering on uniform terrains. This study introduces a kirigami-based robotic skin possessing 3 key innovations: (a) the multistability of the design enables large elongations (>50%) with only small variations in the activation force; (b) the structural design contributes friction anisotropy for improved locomotion; and (c) through gradual unfolding, uniform deformation without distortion or crumpling is obtained.

## Materials and Methods

### Design of the antagonistic fiber-reinforced inflatable actuators

Antagonistic actuators are paired actuators that produce opposing forces to enable controlled, muscle-like movement. The robot’s movement relies on 2 pairs of antagonistic, fiber-reinforced inflatable actuators made of silicone rubber, each featuring 2 axial chambers for linear expansion and bidirectional bending (see Fig. 1C). These actuators are connected via a 3D-printed PLA (polylactic acid) connector. The fabrication is performed with a stereolithography (Form 3, Formlabs) 3D-printed mold comprising 4 parts that shape the internal and external structures and guidelines for fiber reinforcement. Prepolymer silicone (Ecoflex 00-50) is injected and cured at 40 °C for 20 min in a convection oven (Binder FD56). After curing, the actuator is removed and placed in a secondary mold for fiber winding using Kevlar threads in a double-helix pattern, allowing controlled linear elongation along its axis without any radial expansion when pressurized. The molded structure is then encapsulated in a plexiglass tube, ensuring concentric alignment, and an additional silicone layer is cast on top to secure the fibers. This is followed by another curing cycle and careful demolding. The actuator is subsequently mounted in a third mold to cast stiffer silicone end caps (Double Elite 32) that match the kirigami skin cross-section, facilitating attachment. After curing at room temperature for 20 min, silicone tubes to enable air injection are affixed to the actuator with silicone adhesive (Sil-Poxy) to create an airtight seal (see Fig. S3).

### Foldable kirigami skin

The kirigami-inspired skin was designed with folds and cuts to introduce friction asymmetry. Existing stretchable kirigami skins used in previous limbless crawlers [23] suffer from unpredictable crumpling and kinking when the underlying actuator bends. Here, the inclusion of folds enhances the flexural compliance of the skin and allows for uniform bending while preserving the asymmetric configuration of overlapping cut patterns. The asymmetric folds produce greater backward friction compared to the forward direction, which is essential for generating the propulsive force needed to move across different terrains. The repeated pattern of kirigami cuts distributes friction forces spatially across the surface, minimizing slippage and enhances locomotion compared to isotropic skins. The folded kirigami skin geometry features a pattern of adjacent rectangles with circular and partial cuts that form flexible expandable hinges (see Fig. 1D). This pattern, replicated horizontally and vertically, mimics the segmented structure of a worm, supporting longitudinal expansion and multidirectional bending. The skin was fabricated using PET (polyethylene terephthalate) film (Mylar, 0.1 mm thickness) for structural integrity and a high-performance laminated textile (Dyneema, 102 g/sqm, 0.13 mm thickness) for durability, cut with a laser cutting machine, and laminated using heat-press techniques. Double-sided adhesive secures the layers, while prefolding and bonding complete the assembly. This customizable design allows fine-tuning of mechanical properties, optimizing the actuator’s flexibility and strength for natural, worm-like motion. The foldable kirigami skin is designed with 20×9 unit cells. Each unicell has a rectangular geometry with an elliptical cut located between the 2 foldable segments. The dimensions of the unit cells are defined as a=6mm, b=4mm, c=8mm, d=16mm, and e=8mm (see Fig. S4 for fabrication steps of the kirigami skin).

### Control architecture

**Fig. 2.**
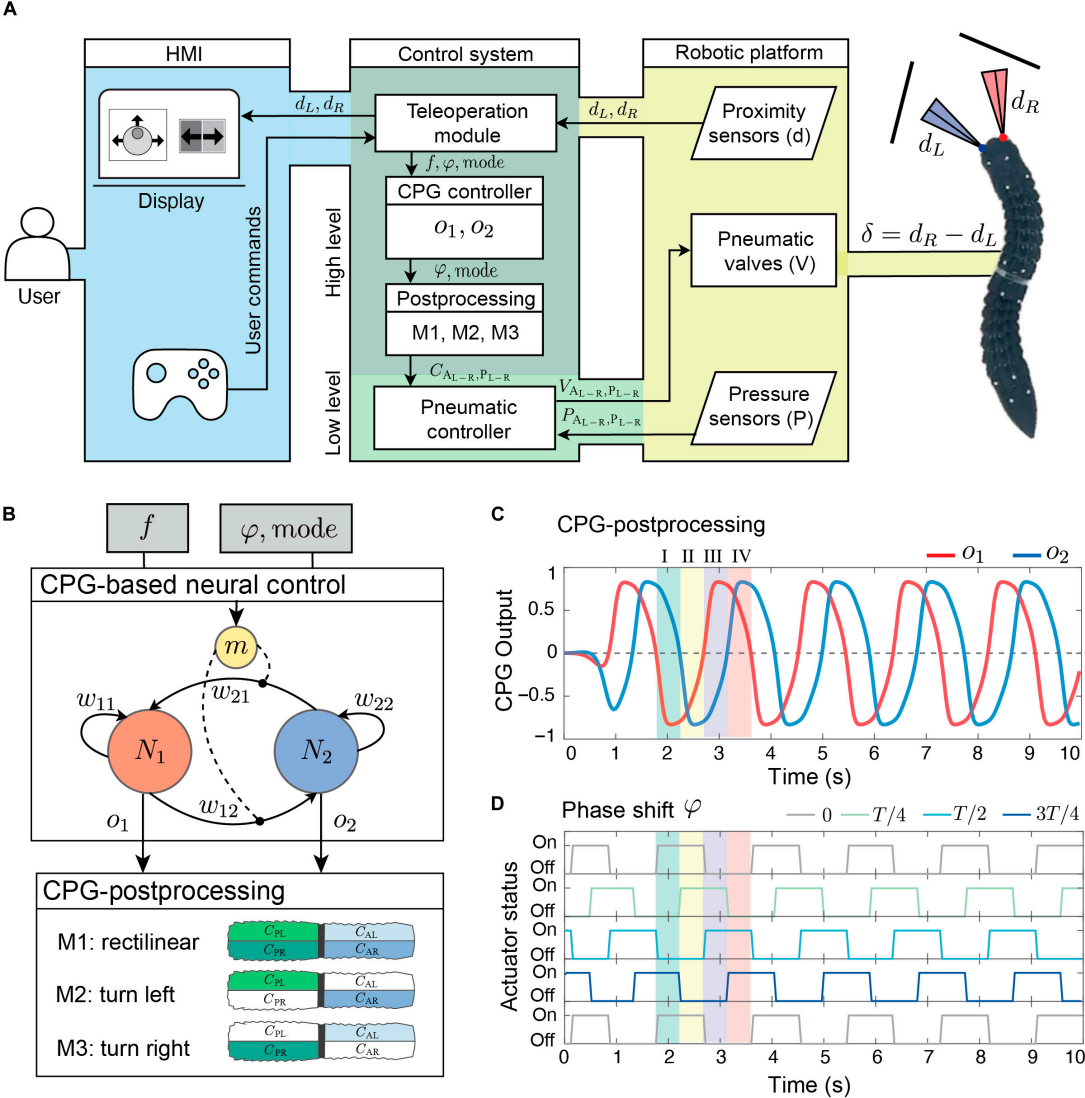
Control architecture. (A) Elements of human–machine interface (HMI), control system, and robotic platform. (B) Structure of the CPG-based controller and postprocessing module. (C) Extraction of discrete regions from the CPG output. (D) Introducing phase shift to the postprocessed signal for controlling pneumatic valves.

The robot’s control architecture consists of 3 main components as shown in Fig. 2A: (a) a human–machine interface (HMI) operated via joystick for assisted teleoperation, receiving user navigation commands and issuing collision alerts; (b) a control block with a teleoperation module, CPG, postprocessing for high-level motion commands, and a pneumatic system for low-level actuation; and (c) the robotic platform with proximity sensors, pneumatic valves, and pressure sensors to collect environmental feedback, control actuation, and monitor pressure.

The teleoperation module translates joystick commands into CPG-based control inputs, interpreting directional commands as specific locomotion modes (e.g., rectilinear, left turn, and right turn). The sensor system continuously monitors obstacles within 20 cm, ensuring safe navigation and overriding user commands to stop or turn when an obstacle is detected within 5 cm. When the distance exceeds 20 cm, the robot moves straight; in the 5- to 20-cm range, differential distance ($\delta =d_{R}-d_{L}$) from 2 sensors triggers steering alerts for right (δ>0) or left (δ<0) adjustments (see Note S3 and Figs. S5 and S6 for more information).

The neural control system incorporates a CPG-based controller paired with a postprocessing module as shown in Fig. 2B. The CPG produces diverse rhythmic locomotion patterns using 2 interconnected neurons, $N_{1}$ and $N_{2}$, with modulatory input m. Each neuron’s behavior was modeled by discrete-time dynamics, where the activity $a_{i}$ at time t+1 depends on synaptic weights $w_{\mathrm{ij}}$ and presynaptic outputs $a_{i}\left(t+1\right)=\sum_{j=1}^{2}w_{\mathrm{ij}}o_{j}\left(t\right),\;i\in \left\{1,\;2\right\}$ [27]. The output follows a hyperbolic tangent function $o_{i}\left(t\right)=\text{tanh}a_{i}\left(t\right)$, with static synaptic weights $w_{11}=w_{22}=w_{0}$ and modulated synaptic weights $w_{12}=w_{1}+m$ and $w_{21}=-w_{1}-m$ that respond to input m. The default synaptic weights are set to $w_{0}=1.3$ and $w_{1}=0.1$ throughout this work. The postprocessing module compares CPG outputs $o_{1}$ and $o_{2}$ dividing the output period into 4 regions noted as I, II, III, and IV [28] for selective activation of specific chambers and generating a wave motion by introducing a phase shift $\varphi =\frac{\mathrm{nT}}{4},n=0,\ldots ,3$ (see Fig. 2C). The resulting signals are assigned to 4 robot chambers ($C_{\mathrm{AR}}$, $C_{\mathrm{AL}}$, $C_{\mathrm{PR}}$, $C_{\mathrm{PL}}$) enabling 3 crawling modes (see Figs. S8 and S9 for more information):

- Rectilinear locomotion: $C_{\mathrm{AR}}\left(I,\;\mathrm{II}\right)=C_{\mathrm{AL}}\left(I,\;\mathrm{II}\right)=1\;\left(\varphi =0\right)$ and $C_{\mathrm{PR}}\left(I,\;\mathrm{II}\right)=C_{\mathrm{PL}}\left(I,\;\mathrm{II}\right)=1$ $\left(\varphi =0,\;\frac{T}{4},\;\frac{T}{2},\;\frac{3T}{4}\right)$
- Turning right: $C_{\mathrm{AR}}\left(I,\;\mathrm{II}\right)=C_{\mathrm{PL}}\left(I,\;\mathrm{II}\right)=1\;\left(\varphi =0\right)$ and $C_{\mathrm{AL}}\left(I\right)=C_{\mathrm{PR}}\left(I\right)=1\;\left(\varphi =\frac{T}{2}\right)$.
- Turning left: $C_{\mathrm{AL}}\left(I,\;\mathrm{II}\right)=C_{\mathrm{PR}}\left(I,\;\mathrm{II}\right)=1\;\left(\varphi =0\right)$ and $C_{\mathrm{AR}}\left(I\right)=C_{\mathrm{PL}}\left(I\right)=1\;\left(\varphi =\frac{T}{2}\right)$.

This control system supports smooth gait transitions, with frequency tuning from f=0.25Hz to f=1.5Hz and phase shifts in T/4 increments. The processed signals trigger on/off valve commands for the pneumatic subsystem, with the control loop running on an Intel Core i7 computer via a Python script.

The pneumatic control subsystem distributes stable air pressure to the robot’s antagonistic soft actuators. Each chamber is independently regulated by a pneumatic solenoid valve (Parker 3-way X-Valve, normally closed) for precise control over inflation–deflation cycles. A silent air compressor (KGK LD-50 L) supplies a constant pressure of P=140 kPa. Power for the valves is provided via a motor driver (Pololu A4988), connected to a microcontroller (ESP32-C6), which also collects data from an integrated silicon pressure sensor (MPX5100, NXP) monitoring each of the 4 chambers. These pressure data ensure safe inflation levels and real-time monitoring. Silicone tubing (1.5 mm inner diameter) connects the actuator chambers to the pneumatic system via barbed and Luer-lock connectors, ensuring secure and reliable airflow.

The robot features 2 distance sensors (Fermion TMF8701 ToF, range: 10 to 600 mm) mounted in a custom 3D-printed PLA head. These sensors, with a 20° field of view, detect obstacles and aid in path navigation. Positioned with an angular offset of 60° from their central detection axes, they provide a lateral detection field for improved environmental awareness. Real-time sensor data are transmitted via I2C to an ESP32-C3 microcontroller, which compiles and sends the data over WiFi to the high-level control loop, facilitating navigation and teleoperation support (see Note S4 and Fig. S7 for more information).

## Results

### Mechanical response of the kirigami skin

**Fig. 3.**
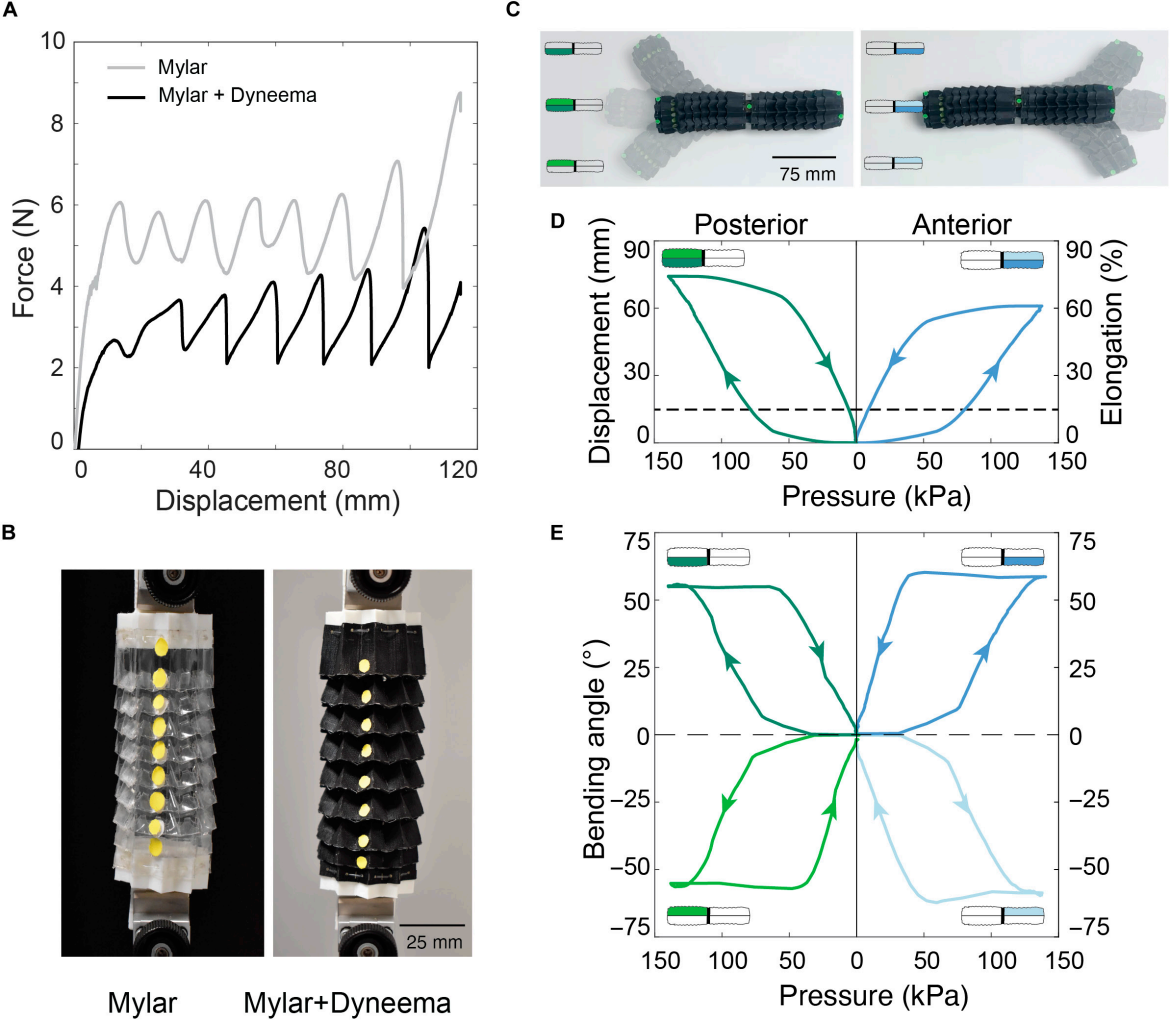
(A) Force–displacement response of the skin for the Mylar layer (gray curve) and a bilayer of Mylar and Dyneema fabric (black curve). (B) Snapshots of the tensile test. (C) Characterization of different actuation modalities. (D) Displacement/elongation and (E) bending angle profiles of the posterior and anterior actuators.

To characterize the force profile of the kirigami skin during robot actuation, we conducted tensile tests using a universal testing machine (Shimadzu EZ Test). We evaluated kirigami skins with 8 rings in 2 configurations: a single-layer Mylar film and a bilayer of Mylar film with Dyneema fabric. Each prototype had an initial folded length of 110 mm and was stretched between 2 clamps at a 0.5 mm/s displacement rate. As depicted in Fig. 3A, both prototypes displayed a similar force–displacement response typical of snapping mechanical metamaterials [29]. The force initially increased linearly, followed by sequential snapping as each fold opened, fluctuating between 4.4 and 6.2 N for the single-layer Mylar and between 2.3 and 4.5 N for the bilayer. Once all folds were open, the force rose steadily. The higher force for the single-layer Mylar resulted from sharp folds that resisted snapping open. With increased fold angles, the bilayer Mylar–Dyneema enabled smoother transitions between states, reducing actuation force. Thus, the bilayer provided a protective layer and facilitated smoother transitions between contracted and expanded states while the snapping behavior limits the force in a controlled range (see Fig. 3B).

### Elongation and bending response of the robot

We characterized the deformation response of the robot to quantify its elongation and bending capabilities (see Fig. 3C). The robot was fixed at the central rigid joint while its deformation during inflation and exhaustion was recorded using a digital camera. Object tracking software tracked the position of markers attached to the actuator tips. The elongation percentage $\varepsilon =100\times \left(l-l_{0}\right)/l_{0}$ is shown in Fig. 3D. The posterior segment elongated up to $\varepsilon_{P}=74\%$, and the anterior segment reached $\varepsilon_{A}=60\%$ at $p_{\max}=140\;\mathrm{kPa}$. Each chamber of the anterior and posterior segments was then independently inflated to the same pressure, and the tip angle was measured. Similar behavior was observed in 4 directions, as shown in Fig. 3E. Maximum bending angles were $\theta_{\mathrm{AR}}=55{}^{\circ}$, $\theta_{\mathrm{AL}}=63{}^{\circ}$, $\theta_{\mathrm{PR}}=57{}^{\circ}$, and $\theta_{\mathrm{PL}}=49{}^{\circ}$. Variations across chambers for both elongation and bending were attributed to fabrication inaccuracies. In both experiments, most deformation during inflation occurred after the pressure surpassed a threshold of $p_{i}\approx 60\;\mathrm{kPa}.$ During deflation, the actuators began to return to their undeformed state when the pressure dropped below $p_{d}\approx 50\;\mathrm{kPa}$. This behavior is attributed to the minimum pressure required to deform the actuator and the inherent hysteresis in the force–displacement response of the multistable kirigami skin during the opening and contraction of folds.

### Friction response

**Fig. 4.**
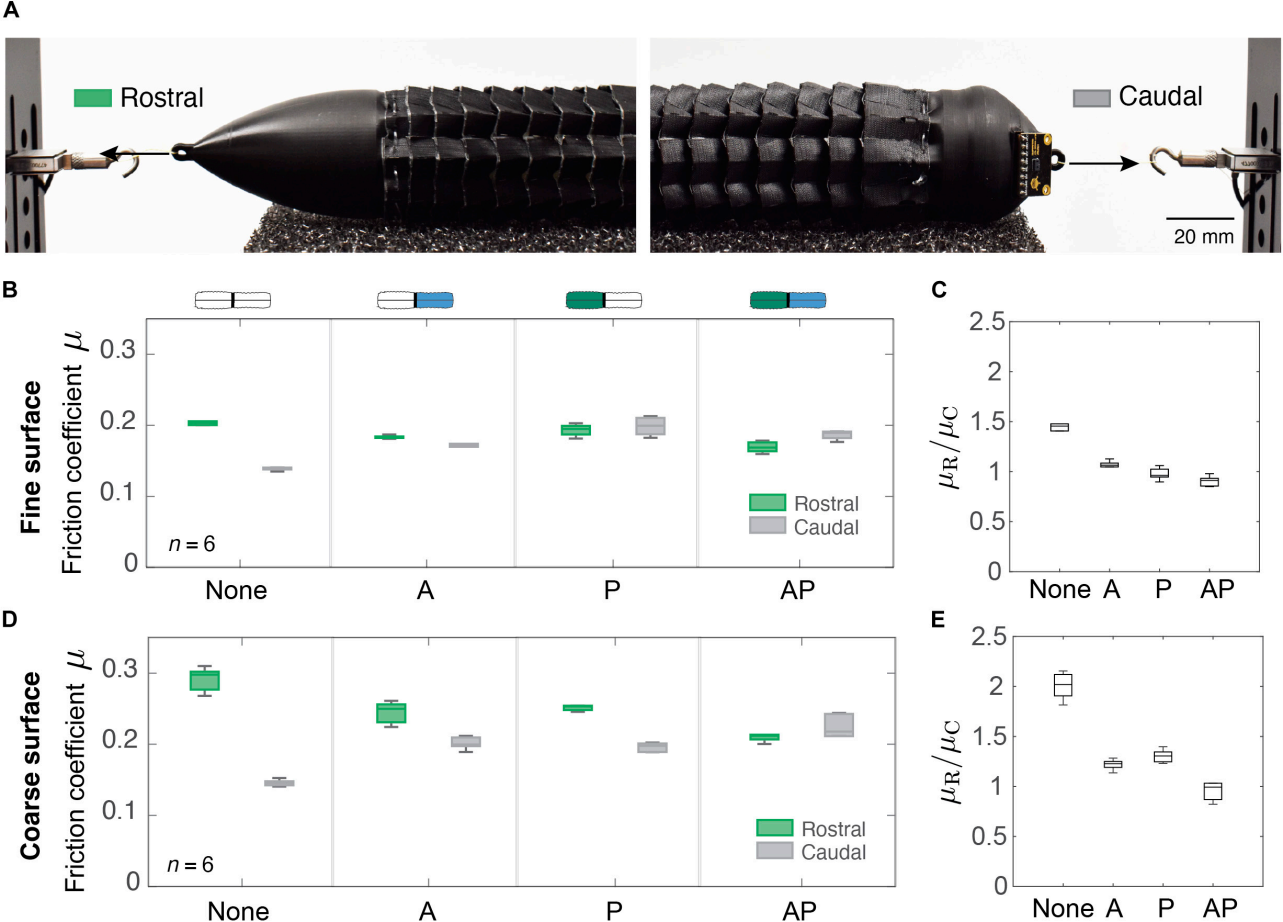
Friction response of the robot. (A) Friction measurement setup for pulling the robot in rostral and caudal directions. (B) Friction coefficients and (C) friction asymmetry ratio of the robot on fine surface (PPI30). (D) Friction coefficients and (E) friction asymmetry ratio of the robot on the coarse surface (PPI10).

We measured the robot’s resistive force when pulled by a Kevlar thread (40 Tex, Aramid) using a motorized linear stage (LTS300C, Thorlabs) against polyurethane foam surfaces with finer (PPI30) and coarser (PPI10) pores with average pore diameters of ø_PPI30_ = 0.85 mm and ø_PPI10_ = 2.54 mm, respectively. Friction force was recorded with a load cell (LSB200 S-Beam 5 lb, FUTEK) mounted on the linear stage as the robot was pulled 200 mm at a constant speed of 10 mm/s. Tests were performed (*n* = 6) for uninflated and 3 inflation states when anterior (A), posterior (P), and both (AP) segments were inflated, with measurements taken in caudal (along scales) and rostral (against scales) directions (see Fig. 4A). Friction coefficients were calculated as $\mu_{C}=F_{C}/W$ and $\mu_{R}=F_{R}/W$ where W=1.4N was the robot’s weight. Inflation of the robot’s segments affected friction force compared to the uninflated state (see Fig. 4B and D).

Overall, we observed increases in the caudal direction and decreases in the rostral direction, however, for most cases $\mu_{R}/\mu_{C}>1$, which ensures the robot does not slide backward when applying propulsive forces (Fig. 4C and E). For some cases, particularly for AP configuration, the friction asymmetry vanishes, i.e., $\mu_{R}/\mu_{C}\approx 1$, potentially impacting locomotion. However, these quasi-static tests may differ from dynamic conditions seen during actual movement, highlighting the need for further assessments involving active actuation to better capture performance under real motion scenarios.

### Tuning actuation frequency by adjusting the CPG modulatory input

The locomotion speed of the robot is intrinsically linked to the inflation and deflation cycles of the antagonistic fiber-reinforced actuators, which follow the oscillation frequency set by the CPG. We mapped the CPG modulatory input parameter m to the desired oscillation frequencies using the pneumatic low-level control system. During this process, we varied m values while monitoring the oscillatory pressure signal with a pressure sensor, from which we extracted the frequency by averaging the period between 10 successive peaks. We identified $f_{\min}=0.1\;\mathrm{Hz}$ as the minimum frequency that permits a maximum tolerable pressure of $p_{\max}=140\;\mathrm{kPa}$. We also determined $f_{\max}=1.5\;\mathrm{Hz}$ as the maximum frequency at which the minimum elongation ε=15% required to initiate the opening of the folds of the kirigami skin based on the results reported in Fig. 3D. Following this tuning process, we identified different m values corresponding to selected frequencies in the range $f\in \left[0.1,1.5\right]\;\mathrm{Hz}.$

### Rectilinear locomotion

**Fig. 5.**
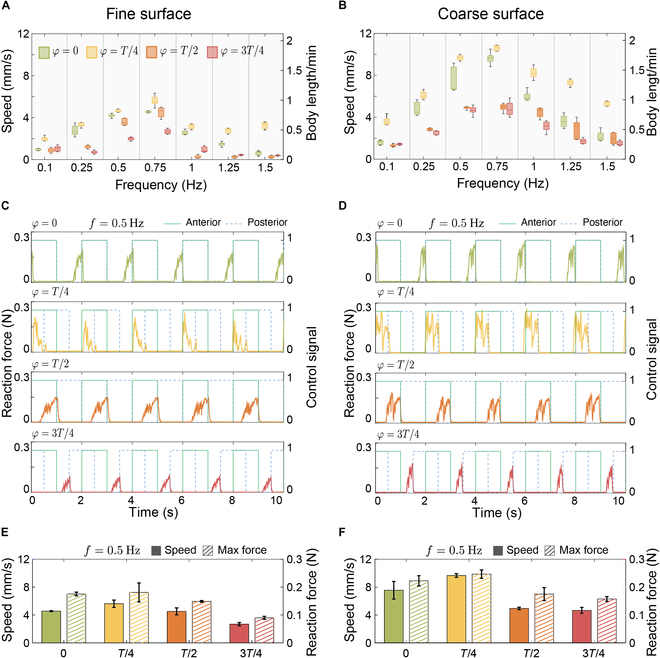
Locomotion speed during rectilinear for different actuation frequencies crawling on (A) fine and (B) coarse surfaces. Pulling force measurements at f=0.5 Hz on (C) fine and (D) coarse surfaces. Correlation of peak forces and corresponding velocity during crawling at f=0.5 Hz on (E) fine and (F) coarse surfaces.

We measured the speed of the robot while crawling in a straight line on both fine and coarse surfaces for a duration of 1 min (see Movie S3). We conducted these rectilinear locomotion tests (*n* = 5) at different phase shifts φ between the actuation of the anterior and posterior segments. The results are summarized in Fig. 5A and B for fine and coarse surfaces, respectively. Overall, the robot achieved faster propulsion on the coarse surface than on the fine surface, due to the improved grip provided by the larger pores. The best crawling performance on both surfaces was achieved for φ=T/4 within the f=0.5−1Hz frequency range (${\bar{v}}_{\max}^{\text{fine}}=6.33\;\mathrm{mm}/s$, ${\bar{v}}_{\max}^{\text{coarse}}=10.83\;\mathrm{mm}/s$). This optimal range reflects a trade-off between body elongation and oscillatory frequency: lower frequencies enable larger displacements per cycle but fewer cycles, whereas higher frequencies yield more cycles with limited per-cycle displacement. Comparing the performance of φ=T/4 and φ=3T/4 reveals that the activation order of body segments plays a critical role in locomotion, despite their similar temporal phase shifts. In the φ=T/4 protocol, the anterior segment activates before the posterior, maintaining frictional engagement in the posterior segment, which supports propulsion by anchoring the body against the substrate. Conversely, in the φ=3T/4 protocol, the posterior segment activates first, followed by the anterior segment, reducing this anchoring effect and diminishing propulsion efficiency.

We developed a simple theoretical dynamic model to qualitatively verify whether the phase shift can influence locomotion speed. The model consists of 3 interconnected masses linked by 2 oscillating elements, each exhibiting variable asymmetric friction with the ground (refer to Note S1 for detailed information). Our numerical results demonstrate that the phase shift plays a critical role in determining the distinct dynamic behaviors across the robot body (see Fig. S1). These behaviors are comparable to the tracked position history of 3 points located at the front, middle, and end of the robot (see Fig. S2). The phase shift substantially alters the friction forces acting on the system, thereby leading to variations in crawling velocities. Specifically, the phase shift modulates the timing and coordination of the oscillating links, which, in turn, affects how the friction forces are distributed and utilized for propulsion (see Movie S2).

### Dynamic pulling force

To gain deeper insight into the linear speed results presented in the previous section, we measured the pulling force exerted by the robot under different phase shifts, φ, while crawling on both fine and coarse surfaces (see Movie S4). All tests were conducted at a frequency of f=0.5Hz. For measuring the robot’s pulling force, we attached it to a load cell (LSB200 miniature high-performance S-Beam, 5 lb, FUTEK) using a Kevlar thread at its posterior end, ensuring the thread was relaxed with no preload (see Fig. S10). The robot was then actuated with 4 phase shifts (φ=0,T/4,T/2,and3T/4) for 2 min. After a few initial cycles, the robot began generating tensile force as it started moving and producing pulling force. This experiment revealed observable differences in both the magnitude and profile of the pulling force across different phase shifts (see Fig. 5C and D). We further analyzed the correlation between the measured reaction force and the robot’s speed. Fig. 5E and F compares the robot’s speed and maximum force averaged over 10 dominant peaks on both fine and coarse surfaces across 4 phase shifts φ (see Fig. S10). The results indicate that pulling forces are consistently higher on the coarse surface than on the fine surface, with the highest forces (${\bar{F}}_{\max}^{\text{fine}}=0.215\;N,{\bar{F}}_{\max}^{\text{coarse}}=0.262\;N$) in both cases observed at φ=T/4. Interestingly, we observed a correlation between the pulling force and the robot’s speed during rectilinear locomotion on both surfaces ($R_{\bar{v}-\bar{F}}^{\text{fine}}=0.77$, $R_{\bar{v}-\bar{F}}^{\text{coarse}}=0.86$). The correlation between the maximum measured force and locomotion performance can be attributed to the static friction at the anchoring limit.

### Steering modalities

**Fig. 6.**
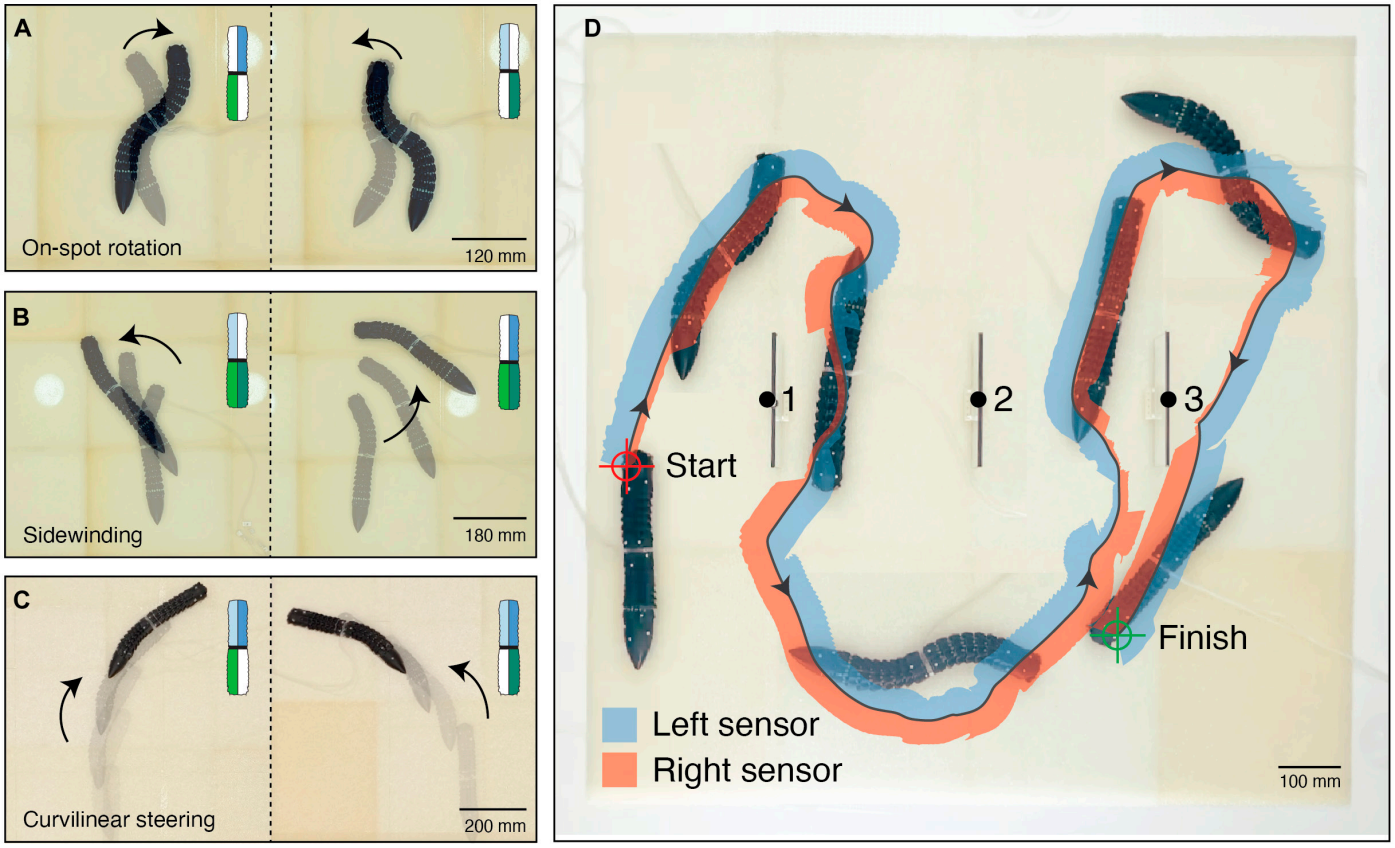
Steering capabilities of the robot. (A) On-the-spot rotation, (B) sideways turning I, (C) sideways turning II, and (D) navigation through obstacles using assisted teleoperation and sensory feedback. The shaded areas indicate distances detected by the proximity sensors, with the values downscaled by a factor of 10 for improved visibility.

To navigate arbitrarily complex paths, the robot is equipped with both rectilinear locomotion and steering capabilities. It changes direction using asymmetric actuation in its anterior and posterior segments. Various actuation combinations can be employed to achieve steering (see Movie S5). For example, cyclic inflation of opposite chambers in the anterior and posterior segments enables on-the-spot rotation at high actuation frequencies, as demonstrated in Fig. 6A. At lower frequencies, similar sequences result in wider turns (see Movie S6). In this gait, activating the right chamber of the anterior segment causes the robot to turn to the right, even if the head is oriented to the left, due to the anterior segment bending to the right. Similarly, activating the left chamber of the anterior segment produces a leftward rotation. In this modality, actuating the opposite chamber in the posterior segment creates a torque that drives the rotation.

Another steering strategy is achieved by actuating 3 chambers. In Fig. 6B, the synchronized posterior chambers and an anterior chamber with a phase shift of φ=T/4 trigger sidewinding toward the activated anterior chamber. In Fig. 6C, 2 synchronized anterior chambers and a posterior chamber with a phase shift of φ=T/4 enable curvilinear steering in the direction opposite to the activated posterior chamber.

### Navigating through obstacles

To further showcase the robot’s capability for limbless crawling through obstacles, an experimental arena was designed. The arena featured a coarse substrate composed of polyurethane foam (PPI10) and included 3 strategically placed obstacles. The robot was tasked with navigating from one side of the arena to the other while avoiding collisions. For this task, rectilinear locomotion with a phase shift of φ=T/4 and a frequency of f=0.5Hz was used for forward progression, while the steering gaits were employed to guide the robot left or right as needed. During the crawling experiment, feedback from onboard proximity sensors was relayed to the HMI, enabling real-time adjustments to the control inputs to prevent collisions. The robot successfully completed the locomotion task, steering through the obstacles and reaching its goal within 18 min (see Movie S7). The trajectory of the robot’s movement, along with feedback from the proximity sensors, is visualized in Fig. 6D.

## Discussion

This study demonstrates that combining asymmetric friction with body deformation in a limbless soft robot enables versatile crawling on flat to moderately rough surfaces. The kirigami-inspired skin enhances surface anchoring through directional friction, providing the necessary grip for propulsion. Experimental results reveal that both oscillation frequency and phase control significantly affect locomotion speed, with optimal propulsion achieved when the robot’s segments are actuated with a phase offset of one-quarter of the total actuation period. Parametric evaluations identified optimal frequency and phase windows that maximize locomotion performance. A dynamic pulling force response study further established a direct correlation between crawling speed and the force exerted by the robot, with higher speeds under similar control conditions producing stronger pulling forces.

The developed prototype offers a promising foundation for advanced robotic applications, with the flexibility to integrate additional sensors and teleoperation systems. These capabilities enable real-time feedback and obstacle avoidance, making the robot well-suited for navigating complex environments and adapting to external disturbances. The findings provide valuable insights for the design of bioinspired crawling soft robots, particularly for tasks requiring maneuverability in cluttered environments, such as field exploration and search-and-rescue operations.

### Future directions

To fully realize the potential of combining body deformation with friction modulation in limbless crawling robots, several key areas demand further investigation:

• Sensor technology: Incorporating more advanced and miniaturized sensors to enable finer environmental perception and closed-loop feedback for robust terrain adaptation.
• Navigation algorithms: Developing and testing adaptive control and path-planning algorithms capable of autonomous operation in unstructured and dynamic environments.
• Material development: Exploring new materials and structural designs for the kirigami skin to increase durability, tunability of frictional properties, and environmental resilience.
• Terrain adaptability: Conducting experiments across a broader range of surface roughness and inclinations to systematically quantify locomotion performance under extreme and variable terrain conditions.
• System integration: Advancing untethered implementations with integrated power sources and wireless communication to support real-world deployment.

## Conclusion

In summary, this work introduces a limbless soft robot that leverages kirigami skin and body deformation to achieve efficient and controllable crawling. Through experimental validation, we demonstrate how tuning actuation parameters influences locomotion performance, and we establish a link between speed and generated pulling force. The robot’s modular architecture supports sensor integration and teleoperation, positioning it as a candidate for deployment in search-and-rescue and exploratory missions. Future improvements in sensory, control, and material domains will further enhance the robot’s functionality and real-world applicability.

## Data Availability

The MATLAB script for the theoretical model is available as part of the Supplementary Materials at https://github.com/SDUSoftRobotics/2025_CBSYSTEMS_Tirado. The datasets generated and analyzed during the study are available from the corresponding author on reasonable request.

## Supplementary Materials

Supplementary 1 Notes S1 to S6 Figs. S1 to S10 Movies S1 to S7
