# Supplementary material for: Multimodal Limbless Crawling Soft Robot with a Kirigami Skin
Source: Cyborg Bionic Syst. 2025 Jun 9;6:0301. doi: 10.34133/cbsystems.0301 (PMC12147391; doi:10.34133/cbsystems.0301)
Supplement: Supplementary 1 — Notes S1 to S6 Figs. S1 to S10 Movies S1 to S7 [file cbsystems.0301.f1.zip › SI_R2.pdf]

Supplementary Information

Multimodal Limbless Crawling Soft Robot with a Kirigami Skin

Jonathan Tirado[1], Aida Parvaresh[1], Burcu Seyidoğlu[1], Darryl A. Bedford[2], Jonas Jørgensen[1], Ahmad Rafsanjani[1,*]

[1] SDU Soft Robotics, Biorobotics Section, The Maersk McKinney Moller Institute, University of Southern Denmark, Odense 5230, Denmark

[2] Drawstring Origami Ltd., London, UK

* Corresponding author ahra@sdu.dk

Date: May 5, 2025

This document contains:

- Note S1. Theoretical model
- Note S2. Fabrication
- Note S3. Assisted teleoperation
- Note S4. System's architecture
- Note S5. Additional results
- Note S6. Description of supporting videos
- Figures S1-S10

Note S1. Theoretical model

We modeled the robot's dynamics with a simple theoretical model consisting of three nodes connected by two oscillating links. The model incorporates directional friction forces, interaction forces between nodes, and gravity to describe the dynamics of the system. Periodic actuation of links generates directional motion. Each node has a mass m_i and moves along a one-dimensional axis. The links between the nodes are modeled as springs with a spring constant k. The lengths of the anterior (A) and posterior (P) links elongate periodically with time, simulating actuation:

$$L_A(t) = L_0 + \frac{A}{2}(1 - \cos(2\pi f t)) \tag{S1}$$

$$L_P(t) = L_0 + \frac{A}{2}(1 - \cos(2\pi f t + \varphi)) \tag{S2}$$

Here, L_0 is the undeformed length of the links, A is the amplitude of elongation oscillations, f is the actuation frequency, and φ is the phase shift between the oscillations of the two links. Each node experiences friction forces that depend on the direction of motion defined by forward friction coefficient μ_f and backward friction coefficient μ_b. The friction force on each node is given by:

$$F_i^{friction} = -\mu_i m_i g \, \mathrm{sign}(\dot{x}_i), \quad i = A, P, M \tag{S3}$$

where $\mu_i = \mu_f \ if \ \dot{x}_i > 0$ and $\mu_i = \mu_b \ if \ \dot{x}_i \leq 0$. Since the friction varies with the reconfiguration of the surface caused by the elongation of each segment, motivated by experiments, we assumed friction coefficients varies linearly with the elongation as

$$\mu_f(L) = \frac{\mu_{f2} - \mu_{f1}}{A}(L - L_0) + \mu_{f1} \tag{S4}$$

$$\mu_b(L) = \frac{\mu_{b2} - \mu_{b1}}{A}(L - L_0) + \mu_{b1} \tag{S5}$$

Where we assumed $L = L_A(t)$ for the anterior node, $L = L_P(t)$ for the posterior node, and $L = (L_A(t) + L_P(t))/2$ for the middle node. The interaction forces between the nodes are modeled as spring forces:

$$F_{PM}^{interaction} = k(x_M - x_P - L_P(t)) \tag{S6}$$

$$F_{MA}^{interaction} = k(x_A - x_M - L_A(t)) \tag{S7}$$

The dynamics of the system are governed by Newton's second law. The equations of motion for the three nodes are:

$$m_P \ddot{x}_P = F_P^{friction} + F_{PM}^{interaction} \tag{S8}$$

$$m_M \ddot{x}_M = F_M^{friction} - F_{PM}^{interaction} + F_{MA}^{interaction} \tag{S9}$$

$$m_A \ddot{x}_A = F_A^{friction} - F_{MA}^{interaction} \tag{S10}$$

The oscillating links (L_A and L_P) transfer energy to the nodes, overcoming friction and enabling motion. The difference between forward and backward friction coefficients (μ_f and μ_b) is crucial for net motion. Higher backward friction ($\mu_b > \mu_f$) ensures that the system moves forward during actuation. The stiffness constant k determines how efficiently the interaction forces propagate through the system. The system exhibits nonlinear behavior due to the sign-dependent friction

forces and the time-varying link lengths. We solved these equations numerically using `ode113` in MATLAB, a numerical solver for solving ordinary differential equations (ODEs) with high accuracy with automatic step-size adjustment.

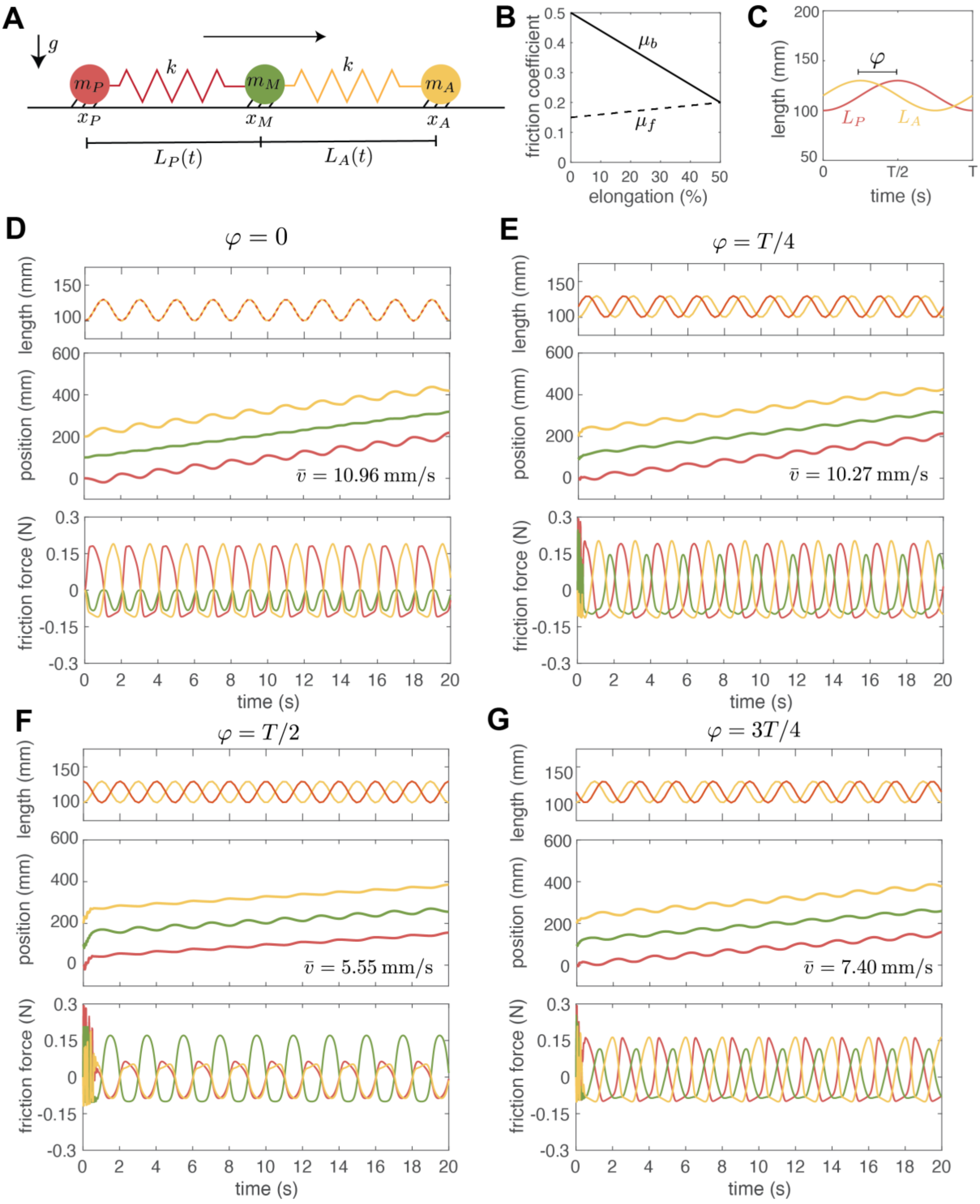

Fig S1. (A) Schematic of the theoretical model for a two-segment crawling robot. (B) Friction coefficient as a function of elongation, (C) definition of the phase shift. Imposed actuation length, calculated position of three nodes, and the estimated friction force on each of them are demonstrated for (D) $\varphi = 0$, (E) $\varphi = T/4$, (F) $\varphi = T/2$, and (G) $\varphi = 3T/4$. The color of all plots is in accordance with the schematic in A.

Fig. S1 shows the results for the following parameters: $L_0 = 100$ mm, $A = 0.3\ L_0$, $m_i = 60$ g, $f = 0.5$ Hz, $k = 100$ N/m, $\mu_{f1} = 0.15$, $\mu_{f2} = 0.2$, $\mu_{b1} = 0.5$, $\mu_{b2} = 0.2$, $g = 9.81$ m/s^2. Here, we assumed a large value for k to ensure the nodes follow the imposed actuation signals $L_A(t)$ and $L_P(t)$ and we increased μ_{b1} compared to experiments to be able to qualitatively reproduce the observed behaviors in experiments. We also assumed the following approximation: $\text{sign}\ x \approx \tanh 50x$ to overcome numerical instabilities.

In all models, there is a transient period after which the response reaches a steady state. We ran the simulations for 20 s (10 cycles) and estimated the average speed over the last five cycles. The numerical results shown in Fig. S1 suggest that the robot actuated with $\varphi = 0$ and $\varphi = \mathrm{T}/4$ outperformed the cases with $\varphi = T/2$ and $\varphi = 3T/4$. These results are in good qualitative agreement with the experiments, as demonstrated by the crawling of the robot on a coarse surface in Fig. S2. Notably, simply by varying φ, the model can reproduce comparable velocities and very similar displacement profiles for the three nodes. Additionally, the model enables estimation of the friction forces between each node and the substrate, revealing that varying phase shifts produce distinct friction force profiles (see the third row in Fig. S1D-G).

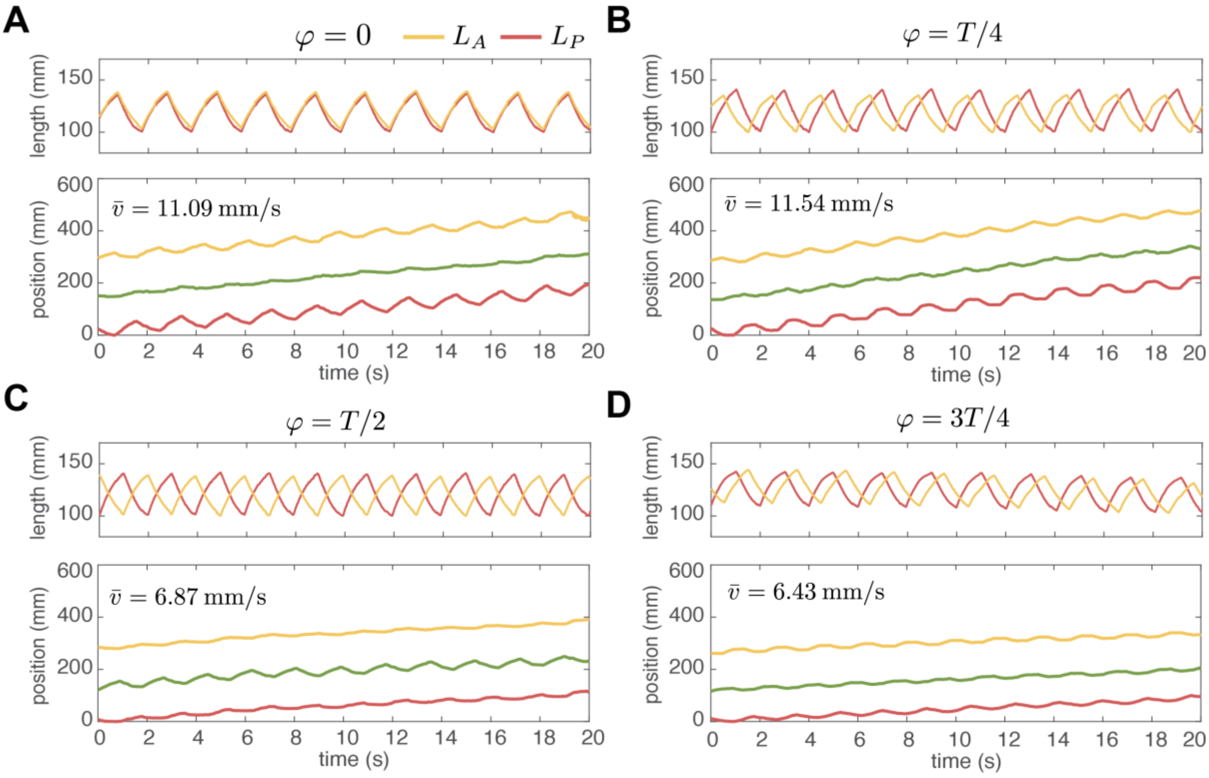

Fig S2. Experimental results for crawling of the robot on the coarse surface (PPI10) for different phase shifts. Actuation length for anterior L_A and posterior L_P segment (top) and tracked position of three nodes (bottom) for (A) $\varphi = 0$, (B) $\varphi = T/4$, (C) $\varphi = T/2$, and (D) $\varphi = 3T/4$.

The MATLAB script: https://github.com/SDUSoftRobotics/2025_CBSYSTEMS_Tirado

Note S2. Fabrication

The kirigami skin is constructed using a geometric pattern of adjacent unit cells, each composed of rectangular segments with elliptical and partial cuts that form flexible, unfoldable hinges. This pattern is repeated vertically to support longitudinal extension and multidirectional bending. Each unit cell consists of a rectangular geometry, where the elliptical cut, defined by dimensions $a = 6\,mm$, $b = 4\,mm$, is located between two foldable segments. The sides of the unit cell are defined by $c = 8\,mm$, $d = 16\,mm$, and the width is specified by $e = 8\,mm$ (see Fig. S4A). The final structure consists of 20 x 9 unit cells, arranged in two halves (10 x 9 units per half) to maintain the symmetry and avoid undesired bending caused by the stiffened overlapping edges (see Fig. S4B).

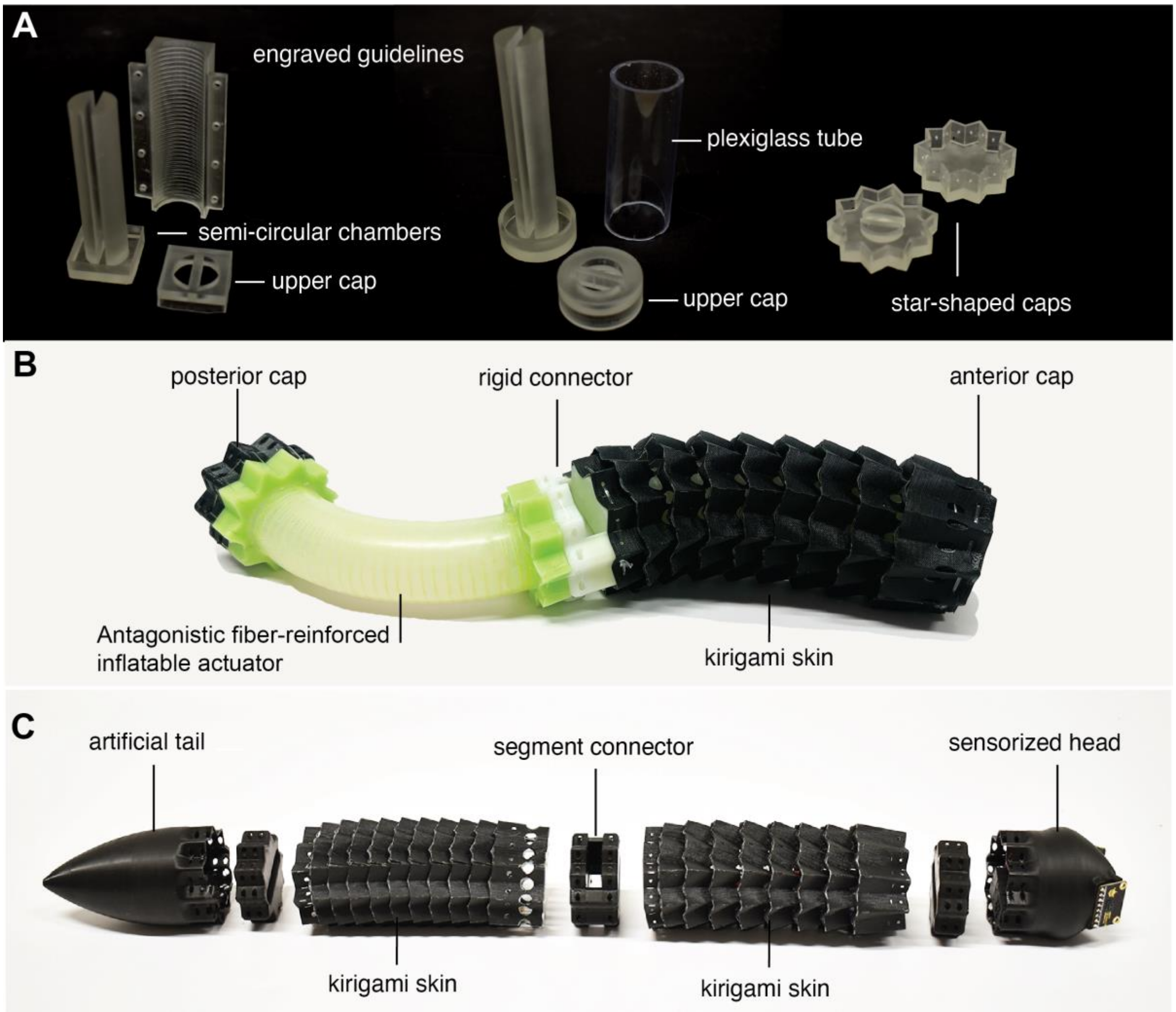

Fig. S3. Design and fabrication of the robot. (A) Fabrication of Antagonistic Fiber-Reinforced Inflatable Actuators: The actuators are fabricated using SLA 3D-printed molds and plexiglass tubes to cast prepolymer silicone. (B) Assembly of the Actuator and Kirigami Skin: The robot is constructed with two antagonistic inflatable actuators joined by 3D-printed segment connectors. The kirigami skin is secured to the actuators using anterior and posterior caps that follow the transversal contour of the skin. These caps feature a series of holes, enabling the use of plastic threads to firmly attach the components. (C) External Elements of the Robot: The robot is complemented by a 3D-printed head and tail. Additionally, two proximity sensors are installed on the lateral sides of the head to detect obstacles and enhance navigation capabilities.

The skin is fabricated by combining PET film (Mylar, 0.1 mm thickness) for structural integrity and a high-performance laminated textile (Dyneema, 102 g/m^2, 0.13 mm thickness) for durability. The geometric design includes cutting lines (red) and folding lines (black) within the unit cell (see Fig. S4A). These features are created using a laser cutting machine to cut (red) and engrave (black) the PET film, the laminated textile, and a double-sided adhesive that bonds the two structural layers (see Fig. S4D-1).

The assembly process begins by laminating the textile layer with one side of the double-sided adhesive using heat press. After peeling the second adhesive side, the PET film is joined using heat, ensuring a one-column shift between the PET film and the textile layer. This shift creates two overlapping columns for closing the kirigami structure (see Fig. S4D2-4). The two halves are then joined by overlaying one of the free columns (see Fig. S4D-5).

Once the full kirigami pattern is assembled, the vertical guidelines are pre-folded to form a sequence of peaks and valleys, creating a zig-zag configuration (see Fig. S4D6). After folding the complete structure, the second free column is joined, forming a ten-point star prismatic body (see Fig. S4D-7). Finally, the horizontal divisions are folded in a zig-zag pattern, creating the segmented hinges characteristic of the kirigami skin (see Fig. S4D-8). The completed structure enables axial extension and contraction, as well as multidirectional bending (see Fig. S4C).

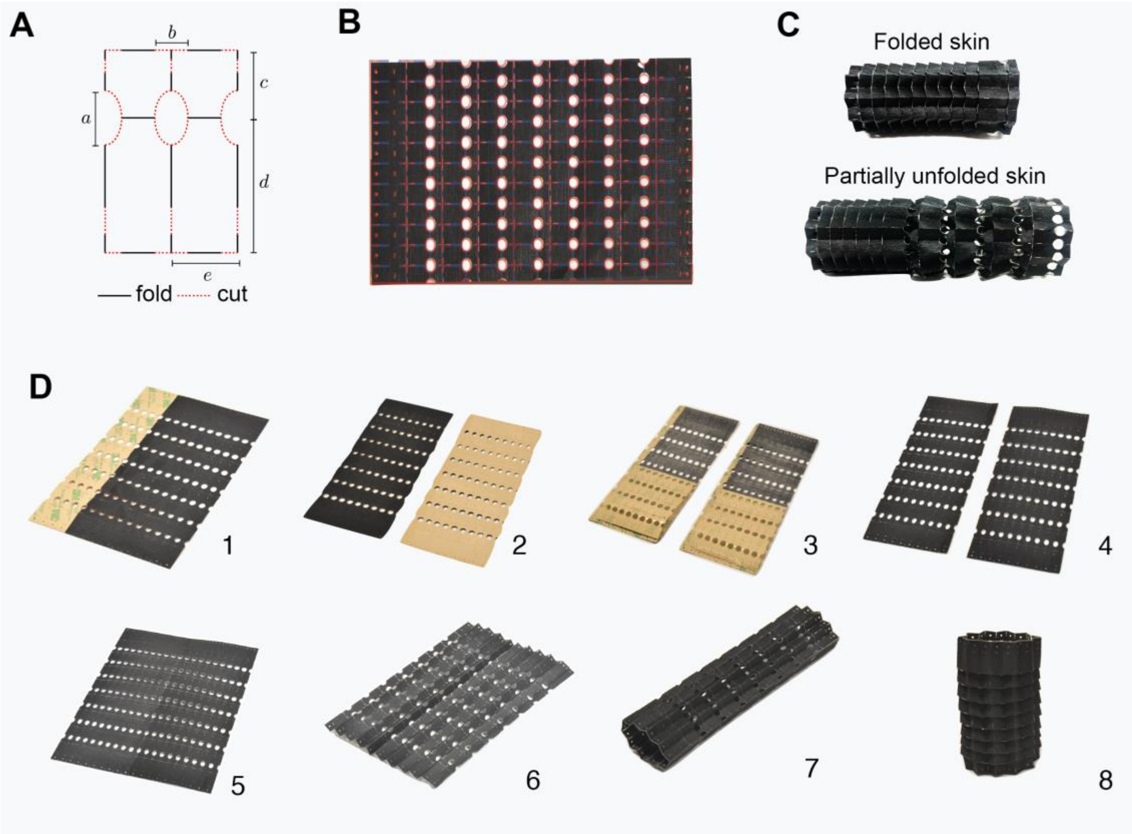

Fig. S4. Fabrication of the kirigami skin. (A) Geometry of the unit cell. (B) laser cut skin in flat configuration. (C) Folded skin in closed (top) and partially open (bottom) states. (D) Fabrications steps for the kirigami skin: (1) Mylar film and Dyneema textile layers are cut using a laser cutter, following the kirigami pattern design. (2-4) The Mylar film and Dyneema textile are laminated together using double-sided adhesive and heat press techniques. (5) The upper and lower sides of the kirigami skin are joined using heat-press techniques for seamless integration. (6) The kirigami skin is pre-folded along the vertical guidelines in an alternating sequence, forming the desired 3D structure. (7) An adhesive layer is applied to securely bond the folded sections of the kirigami skin. (8) Finally, the horizontal guidelines are folded to form flexible, expandable hinges of the kirigami skin.

Note S3. Assisted teleoperation

The assisted teleoperation platform allows a human operator to navigate the crawler robot while receiving real-time proximity sensor data through a graphical interface on a display shown in Fig. S5.

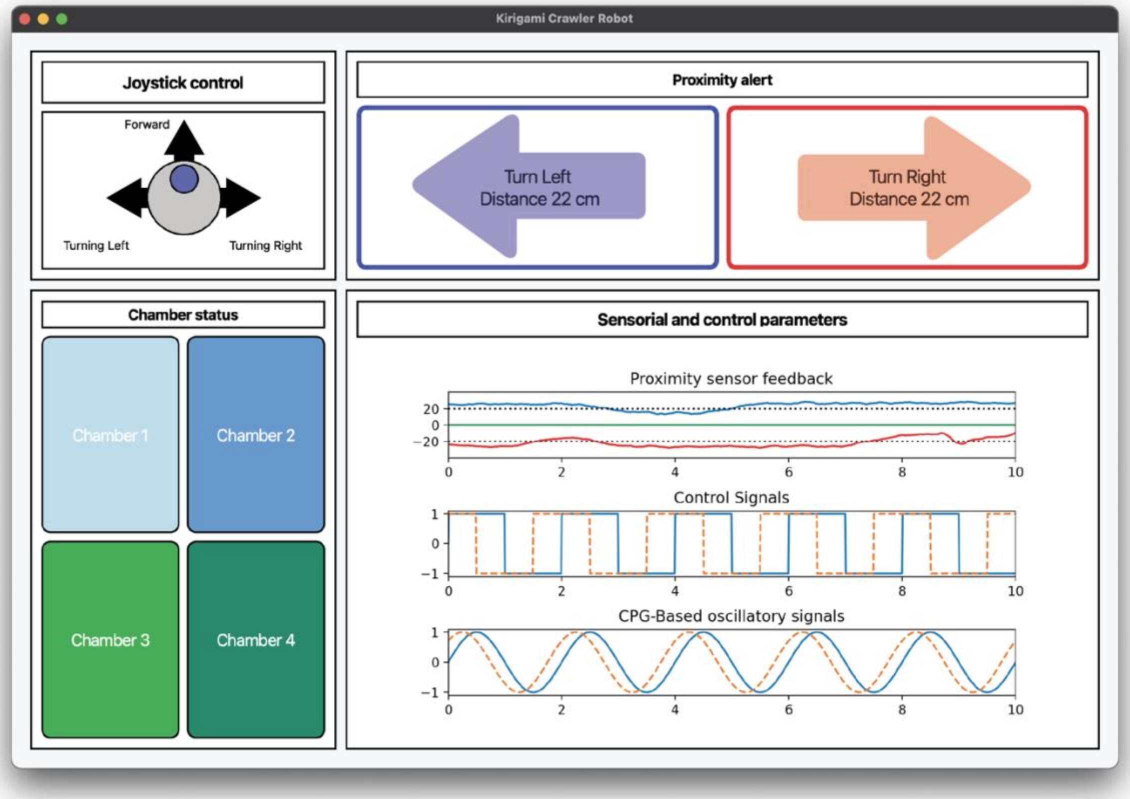

Fig. S5. Snapshots of the display used in assisted teleoperation

In addition to user control, the system incorporates a simple obstacle avoidance logic to enhance navigation safety as demonstrated in Fig. S6B.

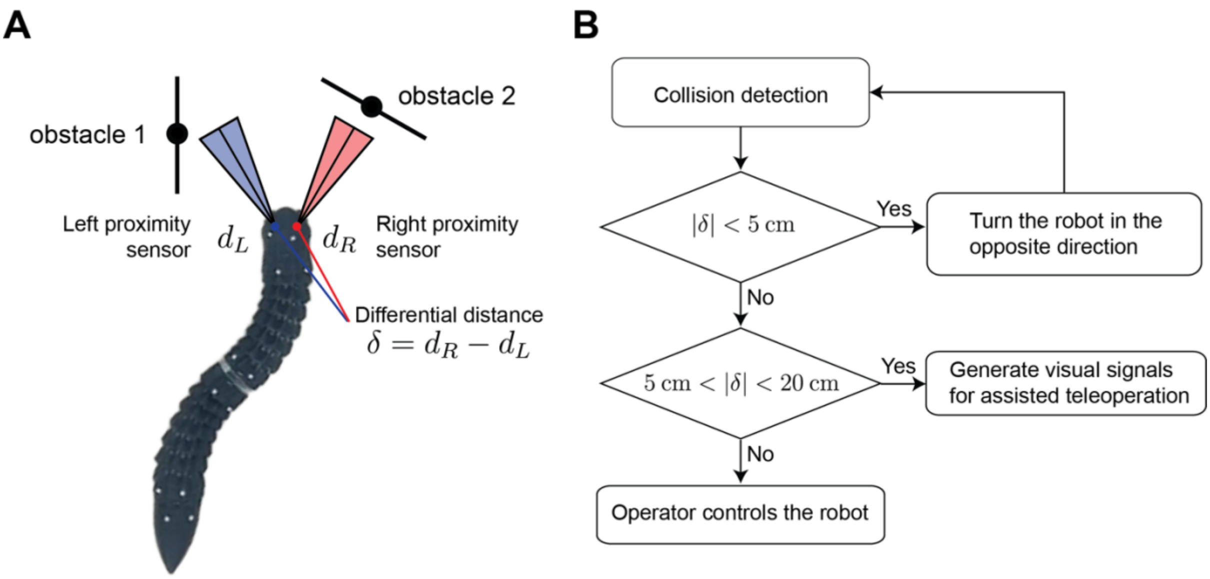

Fig. S6. Assisted teleoperation system. (A) Definition of the differential distance from sensor measurements (B) The algorithm for collision detection.

The user interacts remotely with the system via a computer display and a joystick. The display provides visual feedback from the left and right proximity sensors, while the joystick transmits navigation commands to the control system. The teleoperation module processes these inputs and converts them into control parameters, including oscillation frequency, phase-shift, and crawling modality, which serve as inputs for the CPG-based control and post-processing module (see Fig. 2). Two proximity sensors are mounted on the robot's head casing that can detect obstacles within a range of 1 cm to 60 cm. The system operates based on three distance intervals to optimize navigation safety: (i) When no obstacles are detected within 20 cm, the operator has full control over the robot's direction; (ii) If an object is detected within the 5 cm to 20 cm range, the system evaluates the differential distance ($\delta = d_R - d_L$) between the two sensors (see Fig. S5A). A positive δ prompts the system to alert the operator to steer right, while a negative δ suggests steering left; (iii) When an obstacle is detected within 5 cm, the system overrides the operator's command and automatically forces the robot to turn in the opposite direction to avoid a collision.

Note S4. System's architecture

Fig S7 shows the integration of different components of the proposed robotic system including the electronic components, pneumatic system, proximity sensors, and teleoperation system.

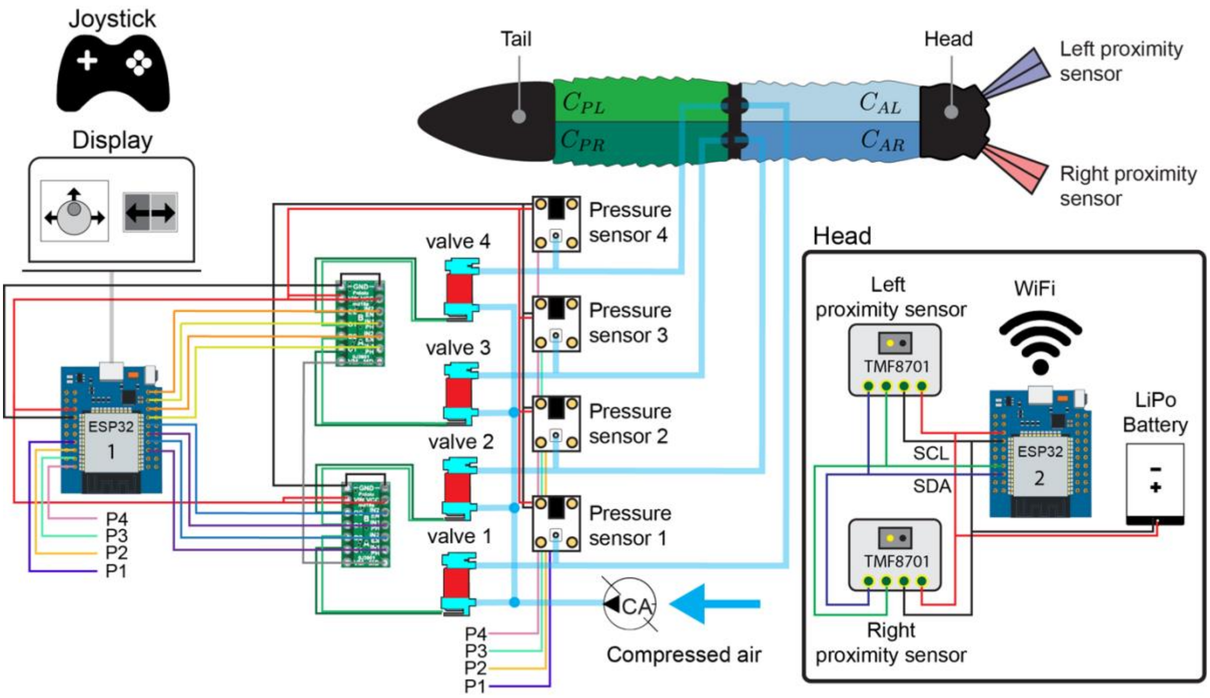

Fig. S7. System's architecture. Schematic diagram of the teleoperation system, showcasing hardware components, electrical and pneumatic connections, and wireless communication.

Note S5. Additional results

In the following we provided additional results on the robot's actuation parameters. Fig. S8 illustrates the output of the central pattern generator (CPG) and the resulting actuation signals for various locomotion modes, including rectilinear movement, sidewinding, and turning. Next, we present the schematic of the robot's actuation sequences for four phase-shifting angles, along with their corresponding pressure responses and variations across different actuation frequencies (see Fig. S9). Additionally, the pulling force exerted by the robot is analyzed over multiple cycles on both coarse and fine surfaces (see Fig. S10).

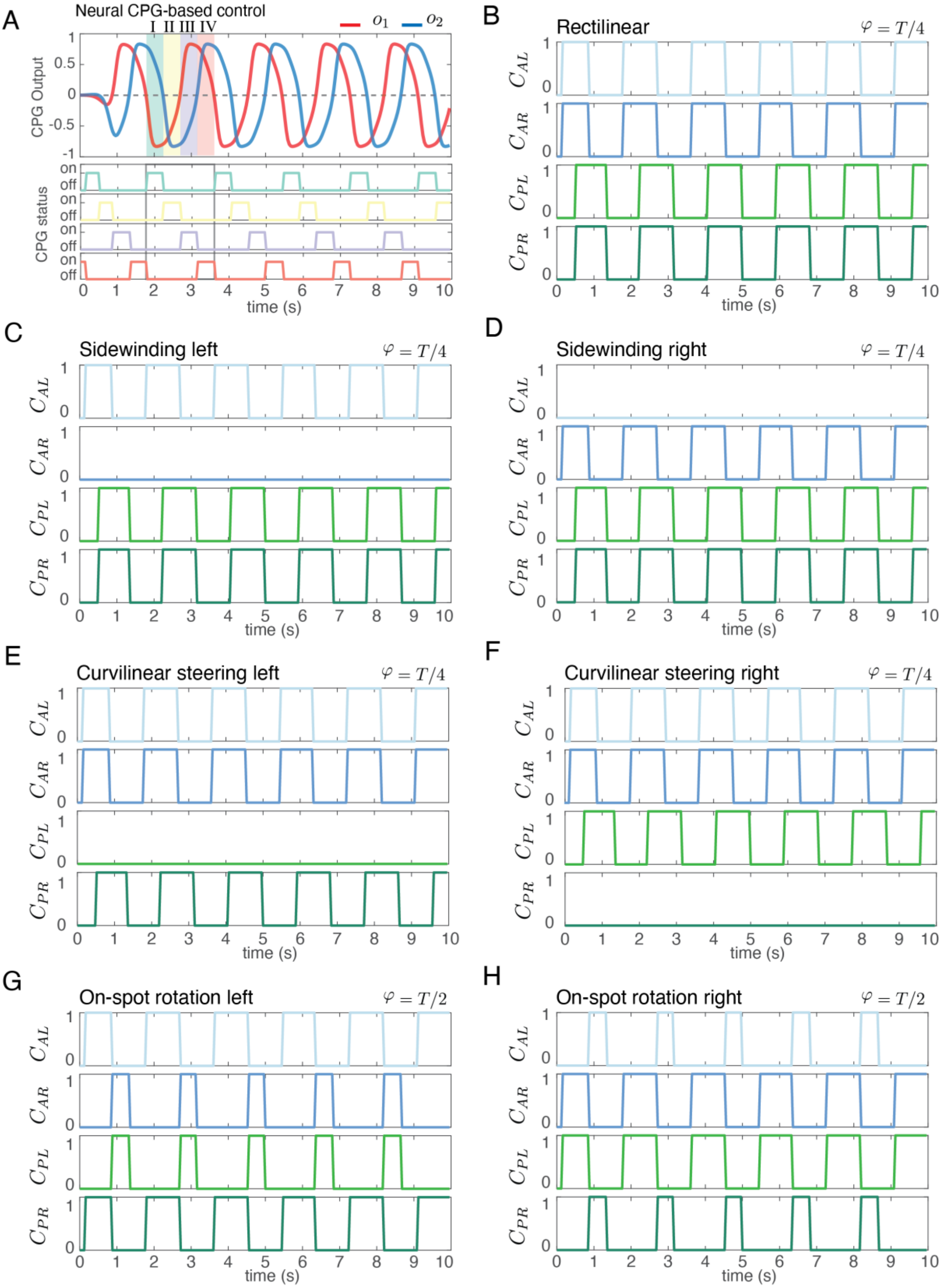

Fig. S8. (A) CPG output and postprocessing. Actuation signals for (B) Rectilinear locomotion, (C) winding I left, (D) winding I right, (E) winding II left, (F) winding II right, (G) turning left, and (H) turning right.

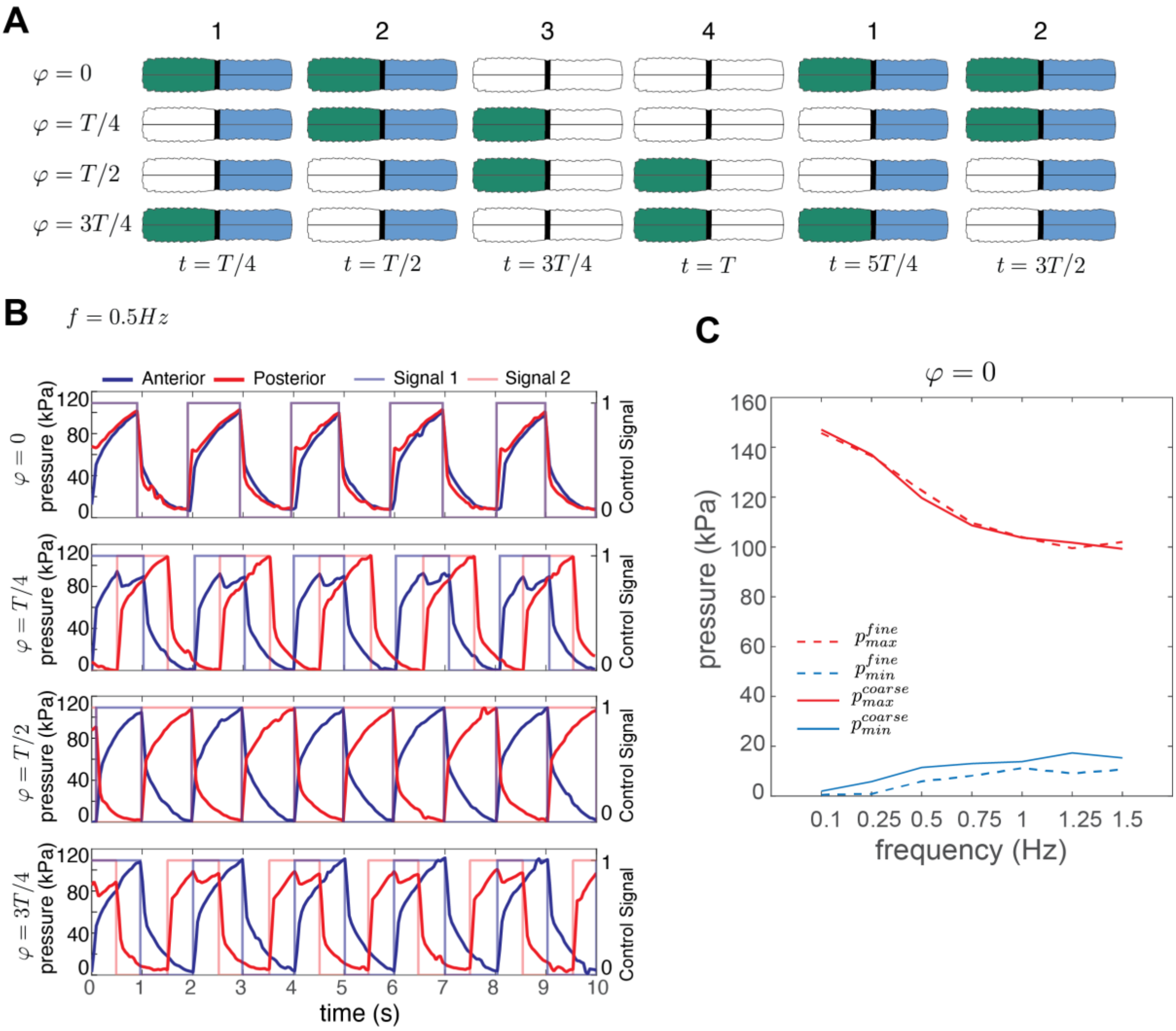

Fig. S9. Robot's actuation sequences. (A) Cyclic activation sequence for rectilinear locomotion with different phase shifts φ. (B) Cyclic pressure profiles for different phase shifts φ. (C) Minimum and maximum pressures at different actuation frequencies.

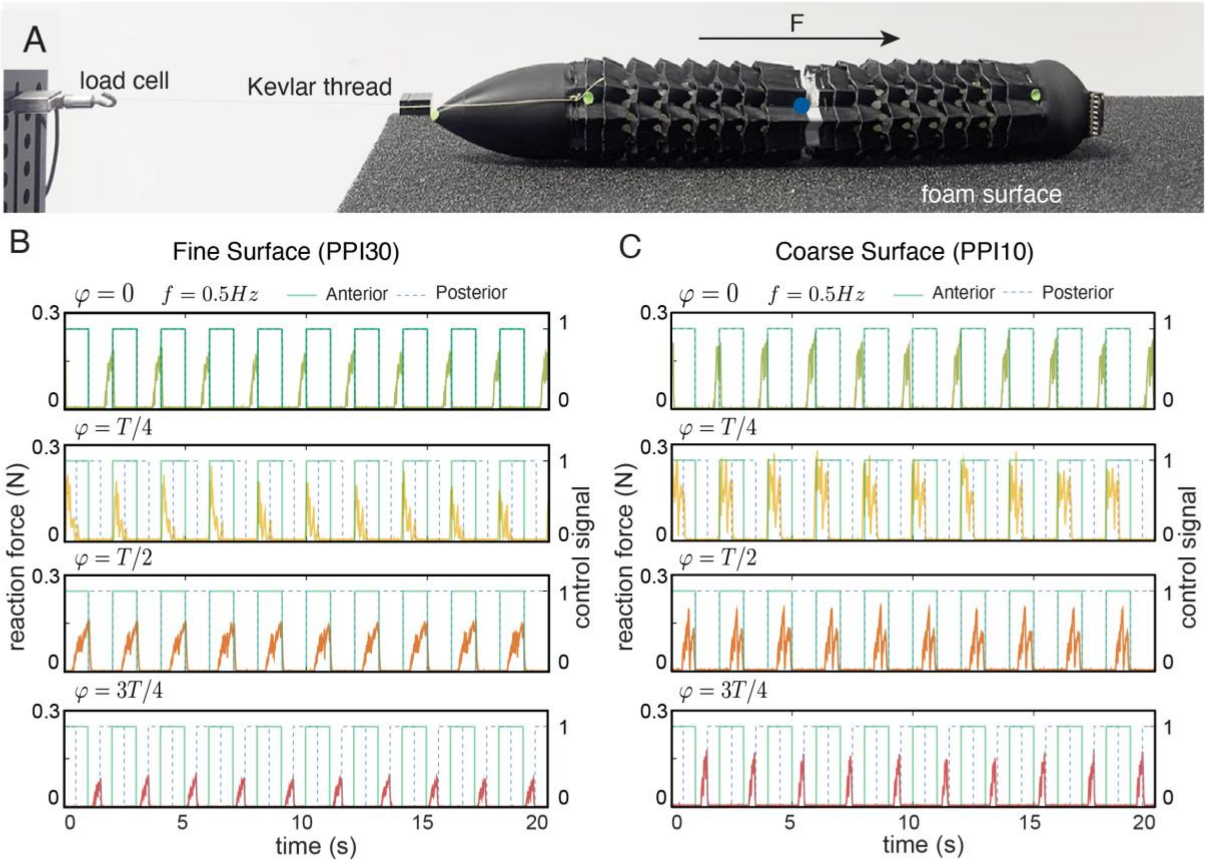

Fig. S10. Measured pulling force during locomotion. (A) Experimental setup for measuring pulling force. Pulling force history for multiple cycles on (B) coarse and (C) fine surfaces.

Note S6. Description of supporting videos

Video S1. Different combinations of actuation sequences of the right and left chambers of the anterior and posterior segments of the robot.

Video S2: Rectilinear locomotion. Theoretical model animation

Video S3: Rectilinear locomotion. Experimental results

Video S4: Traction measurement experiment on coarse and fine surfaces

Video S5: Steering modalities for on-spot rotation and sideways turning.

Video S6: Complete rotation around a circular path.

Video S7: Steering through obstacles with assisted teleoperation and feedback from proximity sensors.
